# Breastfeeding Practices for COVID-19-Infected Mothers: A Systematic Review and Meta-Analysis

**DOI:** 10.3390/nursrep14010040

**Published:** 2024-02-27

**Authors:** Maria Eleni Boukoura, Maria Dagla, Kleanthi Gourounti, Alexandra Stavroula Nieri, Chrysoula Taskou, Eleni Tsoukala, Antigoni Sarantaki

**Affiliations:** 1 Midwifery Department, Faculty of Health and Care Sciences, University of West Attica, Egaleo, 122 43 Athens, Greece; marilena1410@gmail.com (M.E.B.); mariadagla@uniwa.gr (M.D.); kgourounti@uniwa.gr (K.G.);; 2 Nursing Department, National and Kapodistrian University of Athens, 157 72 Athens, Greece; 3 IASO Maternity Hospital, 151 23 Athens, Greece

**Keywords:** COVID-19, coronavirus, SARS-CoV-2, breastfeeding, maternal practices, skin-to-skin contact, rooming-in

## Abstract

(1) Background: The ongoing COVID-19 pandemic has led to an increasing number of women giving birth while also grappling with SARS-CoV-2. The objective of this review is to examine the possibility of transmission of the virus from mother to infant through breastfeeding, skin-to-skin contact, and rooming-in and to explore methods for managing COVID-19-positive mother–infant dyads. (2) Methods: A comprehensive search strategy was employed that covered pertinent studies from the Cochrane Library, PubMed Central, and Scopus databases. The Matrix Method and PRISMA guidelines were utilized by the researchers, with the search being updated until 20 December 2021, one year after the initial vaccine delivery. The inclusion criteria for the study involved articles published in English, those employing broad search terms, and those comprising full-text reviews. Additionally, the researchers required that the articles be published from December 2019 onwards. To further analyze the data, a meta-analysis was performed to estimate the rate of infant infection from mothers who engaged in breastfeeding, skin-to-skin contact, and rooming-in practices. (3) Results: Eighteen studies were analyzed in this review, with an infected infant rate of 2.8%. The maternal practices used in these studies ranged from direct separation of the infant to direct skin-to-skin contact, rooming-in, and exclusive breastfeeding. One study investigated the factors associated with positive test results in newborns and found that only the maternal social vulnerability index >90 was a significant predictor. The type of delivery, rooming-in, and the mother’s symptom status were not associated with positive neonatal outcomes. (4) Conclusions: According to current data, the incidence of perinatal infection with SARS-CoV-2 is relatively low. It is advised that mothers adhere to several supportive care measures, including engaging in breastfeeding, skin-to-skin contact, and rooming-in. These measures ought to be complemented by diligent hand hygiene, the wearing of masks, and the cleansing of breasts solely when necessary.

## 1. Introduction

The causative agent of coronavirus disease 2019 (COVID-19) is SARS-CoV-2, and it was first identified in Wuhan, China, in December 2019. This disease can present without any symptoms, or it can range from mild respiratory infection to severe cardiac complications, including life-threatening respiratory infection [1]. The risk of contracting this infection is present at any age and could lead to severe outcomes, including fatality, for those who become diseased [2]. The virus can be transmitted from one person to another when an infected individual speaks, sneezes, or coughs, and a healthy person may inhale these droplets. Still, it is important to note that individuals who come into contact with surfaces or personal items that have been contaminated by an infected person and subsequently touch their eyes, nose, or mouth may be at risk of contracting COVID-19 [3].

Pregnant women and young children are susceptible to COVID-19 [4]. Newborns are more vulnerable to SARS-CoV-2 infection due to the absence of antibodies against coronaviruses [5]. The mode of transmission and potential effects of COVID-19 on the pregnant population and their newborns are ambiguous. The data are still insufficient regarding whether women with COVID-19 can transmit the virus to their newborns during labor and breastfeeding [6]. Thus, the guidelines for taking care of mother–newborn dyads have changed significantly during the pandemic [7]. The concern of possible transmission of the virus from an infected mother to her infant led to the disruption of some practices recognized as crucial for maternal bonding and breastfeeding initiation, such as skin-to-skin contact and rooming-in [8].

Despite these disruptions, the significant advantages of breastfeeding, which are underscored by its nutritional, immunological, and other benefits, highlight the importance of finding ways to maintain this practice safely, even in challenging circumstances. Breastfeeding is a widely acknowledged practice yielding substantial benefits for both children and mothers. Breastmilk is rich in nutrients, bioactive molecules, antibodies, and microorganisms, which contribute to infants’ growth and development, immune maturation, organ development, and microbial colonization [9]. The active and passive immunity provided by breast milk plays an important role in strengthening the infant’s response to infectious diseases [10]. Research indicates that breastfed infants exhibit a reduced likelihood of developing obesity and demonstrate enhanced cognitive abilities, as evidenced by higher IQ test scores [11]. Mothers, in turn, experience advantages such as a decreased risk of breast and ovarian cancer as well as type 2 diabetes [12]. Conversely, limited breastfeeding has been associated with an elevated risk of postpartum depression [12]. Additionally, studies underline the significance of skin-to-skin contact and rooming-in for the mother–infant dyad, which emphasize benefits ranging from facilitating breastfeeding initiation to stabilizing infant glucose levels and maintaining newborn temperature [13].

This study aims at a rigorous exploration of the breastfeeding practices among mothers affected by COVID-19, with a particular emphasis on scrutinizing the potential transmission of SARS-CoV-2 via breast milk. Preceding investigations have consistently illuminated the presence of viral RNA in human milk, juxtaposed against an absence of detectable infectious virus and a conspicuous dearth of evidence supporting the viral invasion of the mammary gland [9]. A paradoxical scenario emerges with the detection of SARS-CoV-2 RNA in the breast milk of infected mothers, where the apparent absence of viable viral particles engenders a nuanced interrogation of the true risk associated with transmission through breastfeeding [14,15,16]. Noteworthy counsel from scientific societies recommends the adoption of precautionary measures—such as spatial distancing and the utilization of personal protective equipment—for SARS-CoV-2-positive mothers during the act of breastfeeding [17].

In light of the discernible heterogeneity in studies of mother-to-child transmission, a rigorous examination of potential ramifications on neonates is deemed imperative, extending beyond the act of breastfeeding to encompass a spectrum of related care practices including the intimate interface of skin-to-skin contact with mothers afflicted by the viral pathogen. Our study sought to investigate the potential for SARS-CoV-2 transmission from an infected mother to her neonate during breastfeeding as well as through skin-to-skin contact and rooming-in. In summation, the principal objective of this study is to address the following pivotal question: What comprehensive effects, if any, manifest in neonates as a consequence of breastfeeding by mothers infected with COVID-19, and how do associated care practices contribute to this intricate narrative?

## 2. Materials and Methods

### 2.1. Design

This systematic review and meta-analysis were conducted following Preferred Reporting Items for Systematic reviews and Meta-Analysis (PRISMA) 2020 standards [18].

### 2.2. Search Strategies

A comprehensive search of PubMed, Scopus, and the Cochrane Library was conducted with a time frame extending up to 20 December 2021, a period of one year following the initial administration of the vaccine. Duplicate entries were subsequently eliminated from the final results. We used the following keywords: “COVID-19”, “SARS-CoV-2”, “Breastfeeding”, “skin-to-skin contact”, “rooming-in”, and “transmission”. These were used both separately and in combination with the help of the Boolean administrators (OR, AND, NOT). Since COVID-19 is a contemporary issue, records published since 2019 were identified in the primary search stage.

### 2.3. Inclusion and Exclusion Criteria of Studies

We used the acronym PICOST to determine the eligibility criteria of the articles and employed it more specifically as follows:

1. Population: The studies included in our analysis involved samples of mothers who had contracted SARS-CoV-2 as well as their respective newborns. SARS-CoV-2 infection had to be ascertained by a nasopharyngeal swab through a molecular test. We excluded studies that included <20 infected mothers;
2. Intervention: We included studies that employed maternal practices such as breastfeeding, skin-to-skin contact, or rooming-in. Rooming-in was defined as allowing the mother and infant to remain together around the clock during the hospital stay. We defined skin-to-skin care as the practice of placing the baby directly on the mother’s bare chest to maximize surface-to-surface contact. Exclusive breastfeeding was defined as providing no other food or drink, not even water, other than breast milk;
3. Comparison: The studies evaluated the transmission of SARS-CoV-2 from mother to infant during various maternal practices;
4. Outcome: The diagnosis of SARS-CoV-2 infection in infants necessitated a nasopharyngeal swab and a molecular test within the initial 30 days of life;
5. Study: We restricted our inclusion criteria to primary quantitative research studies, specifically those that utilized prospective observational studies, retrospective observational studies, intervention studies, and descriptive studies. On the other hand, we excluded secondary studies such as systematic reviews and meta-analyses as well as qualitative studies, letters to the editor, editorials, protocols, and opinion articles. Additionally, we excluded any studies where the full text was not accessible;
6. Timely: We included studies that were published up to 20 December 2021, which was one year after the first vaccine delivery, and were written in the English language. We excluded studies that were written in a language other than English or did not have full-text availability.

### 2.4. Search Outcomes and Data Extraction

The selection of studies for inclusion in the analysis was carried out by two independent researchers who separately screened the titles and abstracts of the retrieved articles. Any discrepancies in opinion were resolved by a third party. The following variables were extracted from the eligible studies: author(s), year of publication, country of origin, number of mothers with SARS-CoV-2 infection, mother’s age, mode of delivery, gestational age, number of infants with and without SARS-CoV-2 infection, and frequency of maternal practices such as breastfeeding, rooming in, and skin-to-skin contact. Additionally, the measures taken by hospitals to prevent the transmission of the virus from mother to infant were also recorded.

### 2.5. Data Synthesis and Analysis

The main outcome measure was the estimate of infected infants born to mothers with SARS-CoV-2 infection. Random-effect models were used. Pooled data were given with 95% confidence interval values (95% CI) and shown by forest plots. We used the I2-index to assess between-studies heterogeneity, and values > 50% were considered as significant. We used a funnel plot and Egger’s test to assess the publication bias, with a *p*-value < 0.05 indicating publication bias. We performed subgroup meta-analyses for rooming-in, breastfeeding, and skin-to-skin contact as the independent variable. Statistical analysis was performed with STATA.

## 3. Results

**Figure 1:**
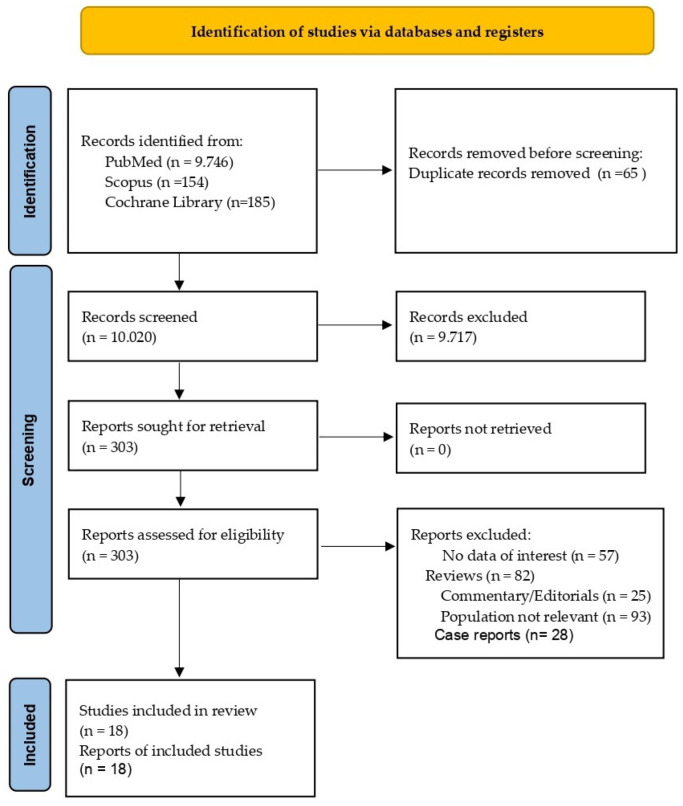
PRISMA 2020 flow diagram for new systematic reviews, which included searches of databases, registers, and other sources.

The initial search was conducted in three databases: PubMed Central, Scopus, and Cochrane Library. A total of 10,085 records were identified through this search. Among the identified records, 9746 were found in PubMed Central, 154 in Scopus, and 185 in Cochrane Library. Overall, 65 records were removed because they were duplicates, leaving 10,020 unique records for further examination. The eligibility assessment involved reviewing the titles and abstracts of the 10,020 records. After this initial screening, 9717 records were excluded, as they were deemed not relevant based on the content of their titles and abstracts. The researchers read the full text of the remaining 303 records that passed the initial screening. Out of the 303 records, only 18 met the criteria for inclusion in the study. The process is visually represented in a flow diagram (Figure 1) following the Preferred Reporting Items for Systematic reviews and Meta-Analysis (PRISMA) guidelines.

From 18 studies, 6 studies were conducted in Spain [19,20,21,22,23,24], 4 each in the USA [25,26,27,28] and Italy [29,30,31,32], and 1 study each in France [33], India [34], Israel [35], and Portugal [36]. Four studies were retrospective [27,31,33,36]. Table 1 represents the main characteristics of the included studies.

Out of a total of 2763 neonates, 79 (2.8%) had a positive COVID-19 test. According to the maternal practices, 54.7% of neonates (440/804) separated early from their mothers [17,23,26,27,29,32], 56.0% (564/1007) neonates were dried and laid directly on the mother’s bare chest after birth (skin-to-skin) [18,19,20,23,30], 66.69% (1199/1798) neonates stayed with the mothers in the same room for 24 h a day from the time they arrived in mothers room after delivery (rooming-in), and 51.5% (971/1886) breastfed exclusively [16,17,21,22,23,24,26,28,29,30,34] (Table 2).

Only one study examined the factors associated with positive SARS-CoV-2 results among neonates [22]. This study showed that the only factor associated with positive SARS-CoV-2 was maternal SVI > 90th percentile vs. other (OR = 4.95; 95% CI: 1.53–16.01; *p* = 0.008). The type of delivery (vaginal vs. cesarean delivery: OR = 0.47; 95% CI: 0.16–1.40; *p* = 0.18), rooming-in (any vs. no: OR = 0.29; 95% CI: 0.04–2.29; *p* = 0.24), and maternal symptoms (symptomatic vs. asymptomatic: OR = 0.71; 95% CI: 0.49–1.02; *p* = 0.07) were not associated with positive SARS-CoV-2 results among neonates [26].

For the spread of the disease, the hospitals took measures such as separating infected mothers from their newborns immediately after birth and during the entire hospitalization [26,32,33], mothers wearing surgical face masks [23,28,30,32,33,34], mothers disinfecting their hands [25,29,30], mothers washing their nipples with odorless soap during breastfeeding [28,33], newborns being placed at least 2 m away from their mother’s bed during sleeping [32,33,34], and the use of personal protective equipment [27].

## 4. Meta-Analysis

**Figure 2:**
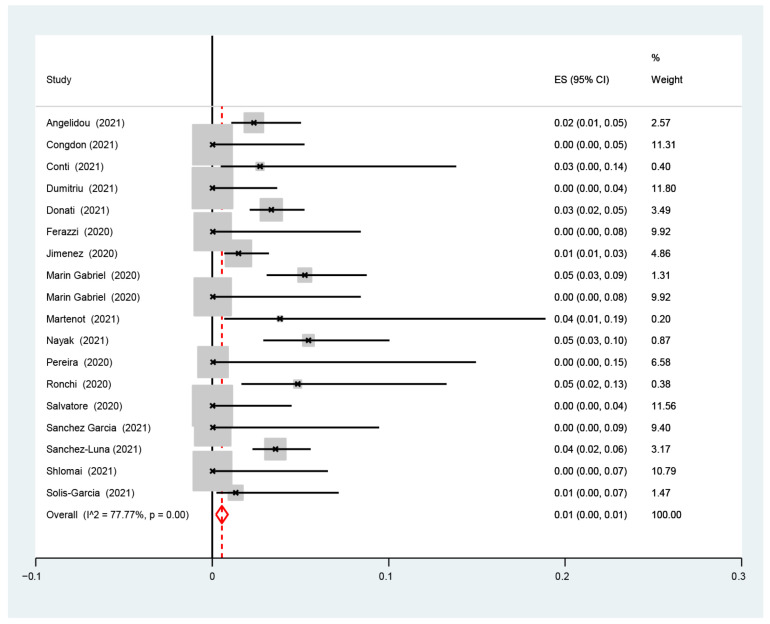
Proportion metanalysis of the overall estimate of SARS-CoV-2 infection among infants born to infected mothers.

The pooled proportion of SARS-CoV-2 infection among infants born to infected mothers was 1.0% (95% CI: 0.0–1.0%). Based on the examination of the available data, it is estimated that 1.0% of infants are born to mothers who have been infected with SARS-CoV-2. The confidence interval suggests that the true proportion is likely to be between 0.0% and 1.0% (Figure 2).

**Figure 3:**
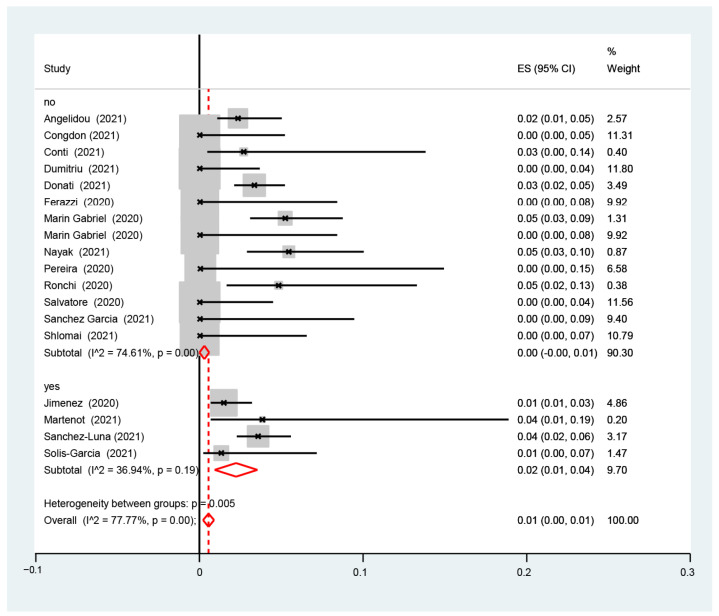
Proportion of the estimate of SARS-CoV-2 infection among infants born to infected mothers in subgroups analyses from studies with and without rooming-in.

**Figure 4:**
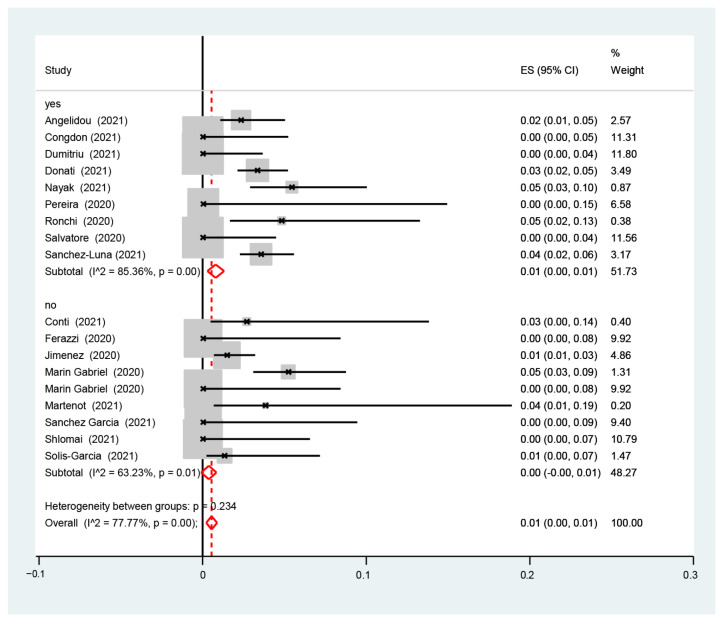
Proportion of the estimate of SARS-CoV-2 infection among infants born to infected mothers in subgroups analyses from studies with and without skin-to-skin.

**Figure 5:**
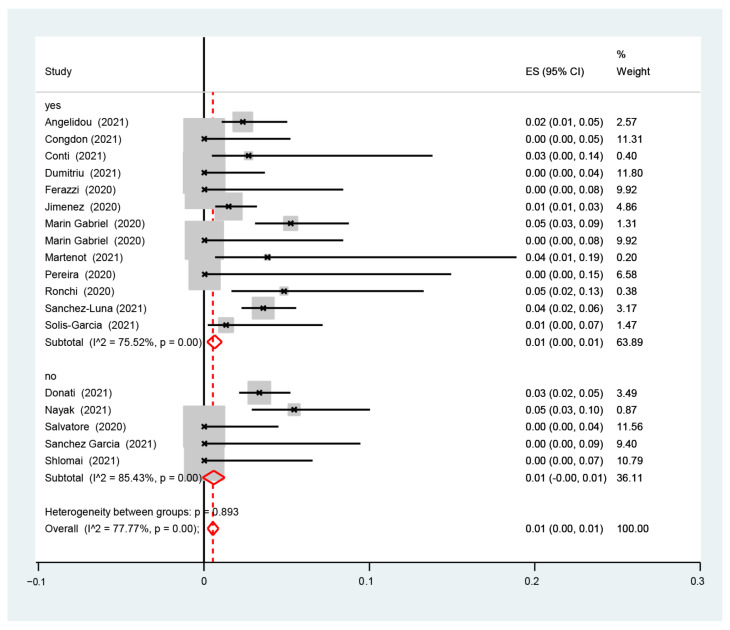
Proportion of the estimate of SARS-CoV-2 infection among infants born to infected mothers in subgroups analyses from studies with and without exclusive breastfeeding.

The I2-test was 77.77%, demonstrating a high heterogeneity. The sub-analysis showed that the proportion of infected neonates was similar between the studies with (1%, 95% CI: 0.0–1%, I2-test 36.94%) and without (0.0%, 95% CI: 0.0–1.0%, I2-test 74.61%) (Figure 3) data for rooming-in, with (1%, 95% CI: 0.0–1%, I2-test 85.36%) and without (0.0%, 95% CI: 0.0–1.0%, I2-test 63.23%) (Figure 4) data for skin-to-skin, and with (1%, 95% CI: 0.0–1%, I2-test 75.52%) and without (1.0%, 95% CI: 0.0–1.0%, I2-test 85.43%) data for exclusive breastfeeding (Figure 5). The red dotted line represents the line of no effect (ES = 0), where values to the left suggest a negative effect, and to the right a positive effect. The red diamond-shaped figure indicates the overall pooled estimate of effect size and its 95% confidence interval, with the horizontal spread of the diamond reflecting the precision of the pooled estimate [19,20,21,22,23,24,25,26,27,28,29,30,31,32,33,34,35,36].

Figure 3 highlights the proportion of SARS-CoV-2 infection among infants born to infected mothers, with a focus on subgroup analyses comparing studies with and without rooming-in practices. The results reveal a statistically significant heterogeneity between the groups (*p* = 0.005), and the effect size (ES) is reported as 0.01 with a 95% confidence interval (CI). The red dotted line indicates the line of no effect (ES = 0). The red diamond-shaped figures represent the pooled effect sizes for subgroups ‘no’ and ‘yes’, with the horizontal spread of each diamond reflecting the precision of these pooled estimates. The plot also displays the subgroup heterogeneity statistics (I˄ and *p*-values).

Figure 4 showcases the proportion of SARS-CoV-2 infection among infants born to infected mothers. The provided figure comprises subgroup analyses derived from studies that categorized infants based on the presence or absence of skin-to-skin contact. The reported heterogeneity between these groups is reported as *p* = 0.234. Forest plot showing effect sizes (ES) with 95% confidence intervals (CI) for individual studies and pooled subgroup estimates. The red dotted line indicates the line of no effect. The red diamond-shaped figures represent the pooled ES for the ‘yes’ and ‘no’ subgroups, with the width of the diamond indicating the 95% CI. Subgroup heterogeneity is quantified with I˄ statistics and corresponding p-values.

Figure 5 depicts the proportion of SARS-CoV-2 infection among infants born to infected mothers. The figure includes subgroup analyses from studies that separated infants based on exclusive breastfeeding and non-exclusive breastfeeding. The reported heterogeneity between these groups is reported as *p* = 0.893. Forest plot of study effect sizes (ES) with 95% confidence intervals (CI). The red dotted line represents the line of no effect. The red diamond-shaped figures indicate the pooled effect size estimates for ‘yes’ and ‘no’ subgroups, with the horizontal tips of the diamonds representing the 95% CI limits. I˄ statistics are provided for subgroup and overall heterogeneity, with their associated *p*-values.

## 5. Discussion

This systematic review and meta-analysis aimed to examine the risk of transmission of SARS-CoV-2 from an infected mother to her neonate during breastfeeding, skin-to-skin contact, and rooming-in. Our findings show that the SARS-CoV-2 infection among neonates born to infected mothers was found to be 1.0%. This indicates that mother-to-child transmission in the neonatal period is low. Moreover, the sub-analysis presented that the proportion of infected neonates was similar between the studies with and without data for rooming-in, skin-to-skin contact, and exclusive breastfeeding.

With respect to breastfeeding, it is commonly acknowledged that COVID-19 is primarily transmitted through horizontal transmission, specifically through aerosolization [37]. In our review, the majority of articles indicated that vertical transmission through breast milk cannot be completely ruled out. There have been studies in which the virus was detected in breast milk, and infants were diagnosed with COVID-19, but the exact mode of transmission was unclear [38]. Nevertheless, the advantages of breastfeeding are deemed to outweigh the potential risks of transmission. Consequently, prominent organizations such as the CDC (Centers for Disease Control and Prevention), WHO (World Health Organization), the American Academy of Pediatrics, and the RCOG (Royal College of Obstetricians and Gynecologists) endorse direct breastfeeding while taking individual protective measures, such as maintaining strict hand hygiene, wearing a surgical mask, and cleaning the breast when necessary (e.g., when the mother coughs or sneezes on the breast) [39].

Notably, breast milk has been discovered to contain anti-SARS-CoV-2 antibodies, which may offer a protective mechanism for newborns [40]. As a rich source of bioactive molecules that promote the immune system development of neonates, breast milk is widely considered to be the ideal source of nutrition for most newborns, even in instances where the mother is infected with COVID-19. Studies have found that breast milk from mothers with COVID-19 contains antibodies and other immunological factors that may provide protection to neonates and infants [41].

Throughout the COVID-19 pandemic and up to the present time, various recommendations for breastfeeding have been implemented. For mothers who are unable to breastfeed immediately or choose not to, it is advised to express milk following protection measures and thorough pump cleaning [42]. In situations where breast milk is not available or insufficient, it is suggested to use pasteurized human milk from milk banks instead of formula [43]. A study by Salvatore et al. [28] examined a cohort of neonates born to SARS-CoV-2-positive mothers and assessed the outcomes of staying with the mother and breastfeeding for up to one month after birth. All newborns in the study tested negative for SARS-CoV-2, either immediately after birth or fourteen days later. This finding indicates that non-separation of the mother–infant dyad and breastfeeding can be safe when appropriate precautions are taken, including hand hygiene and the use of surgical masks [28].

The disruption caused by the early separation of the dyad had a detrimental impact on the percentage of women who were able to successfully breastfeed. Although a modest recovery in the overall breastfeeding rate was registered following the reunification of mother and infant, this improvement was likely attributable to the reduced breastfeeding support that was available during the pandemic [44]. According to the World Health Organization, it is advisable to breastfeed infants and young children if their mothers have suspected or confirmed SARS-CoV-2 infection. The advantages of breastfeeding, which encompasses the practice of skin-to-skin care and the transmission of protective maternal antibodies through breast milk, particularly secretory immunoglobulin A (sIgA) and, to a lesser extent, IgM and IgG isotype immunoglobulins, are well documented [45]. Recent evidence indicates that breastfeeding does not seem to be associated with neonatal SARS-CoV-2 infection because viral transmission through the milk, if any, is rare, and a robust sIgA-dominant SARS-CoV-2 antibody response is detectable in human milk soon after infection in a significant majority of individuals [46].

The findings of this systematic review and meta-analysis provide valuable insights into the risk of SARS-CoV-2 transmission from infected mothers to their neonates, particularly during breastfeeding, skin-to-skin contact, and rooming-in. The identified low prevalence of SARS-CoV-2 infection among neonates born to infected mothers (1.0%) suggests a relatively low risk of mother-to-child transmission during the neonatal period. While the sub-analysis revealed consistent proportions of infected neonates across studies with and without available data, it is essential to acknowledge the limitations of this study and how they may impact the practical implications of the findings. Firstly, the uncertainty surrounding the exact mode of SARS-CoV-2 transmission through breast milk poses a significant limitation. Although vertical transmission through breast milk cannot be ruled out, the known benefits of breastfeeding outweigh the potential transmission risk. However, the lack of clarity on the exact mode of transmission emphasizes the need for ongoing research to elucidate the mechanisms involved. Furthermore, the presence of anti-SARS-CoV-2 antibodies in breast milk is highlighted as a potentially protective factor. While this is promising, the specific role and efficacy of these antibodies in conferring immunity to neonates require further investigation. Future research should delve into the nuances of these antibodies, their duration of effectiveness, and whether they provide comprehensive protection against SARS-CoV-2 in infants. The dynamic nature of the COVID-19 pandemic and the rapid evolution of recommendations for breastfeeding practices introduce a temporal limitation to this review. As the data are subject to change, the suggestions provided in this review may become outdated. It is crucial for future research to continually monitor and update recommendations based on emerging evidence and changes in the aftermath of the pandemic landscape.

The study period concluding a few months after the introduction of the first vaccine raises the need for additional research on women who tested positive for COVID-19 in the early postpartum period and were vaccinated during or before pregnancy. This gap in knowledge underscores the importance of investigating the impact of vaccination on breastfeeding practices, the transfer of immunity through breast milk, and the overall health outcomes for both mothers and infants. Furthermore, the exclusion of pregnant and lactating women from early COVID-19 vaccine trials due to safety concerns represents a substantial limitation. The lack of data on vaccine safety and efficacy for this population emphasizes the need for dedicated research to guide vaccine decision making. In future vaccine trials, it is crucial to include pregnant and lactating individuals. This inclusion should be coupled with a thorough evaluation of immune transfer through both the placenta and breast milk. In order to offer well-founded recommendations, it is crucial to carry out extensive evaluations.

The present study serves as an essential scholarly contribution to the ongoing COVID-19 discussion, despite its recognized constraints, by offering a well-informed and insightful search into pandemic management for breastfeeding mothers, thereby demonstrating its relevance to the field. The insights presented in this study underline the significance of addressing the shortcomings and constraints in research pertaining to pregnancy, breastfeeding, and immunization to facilitate knowledgeable and efficient public health initiatives. As the scientific literature emphasizes the ongoing need for research and counseling on breastfeeding techniques for mothers with COVID-19, it is clear that discrepancies persist, necessitating further investigation to establish optimal health safeguards for both mothers and infants. Amid the complexities of the global healthcare landscape during and after the COVID-19 pandemic, staying abreast of the latest evidence and professional advice is crucial for making informed decisions on breastfeeding practices in the post-pandemic era [47,48,49,50,51,52,53,54,55,56,57].

Balancing the preservation of maternal and neonatal well-being during infectious disease outbreaks while considering the potential repercussions of excessive caution on the emotional and developmental health of the child is of paramount importance. This imperative extends to both the immediate and post-outbreak periods, underscoring the need to strike a judicious equilibrium. Such a balance necessitates a comprehensive approach that carefully weighs the protective measures implemented for health preservation against potential adverse effects on the emotional and developmental facets of the child’s well-being [58]. A more recent study contributed further information to the existing body of knowledge, suggesting that the likelihood of neonates acquiring COVID-19 from their mothers is relatively moderate [59]. However, separating the two individuals, even with the intention of safeguarding the neonate, may have repercussions for the development of emotional attachment and bonding between them [60,61]. The formation of a strong emotional bond between a mother and her child is crucial for the baby’s holistic development. Activities such as skin-to-skin contact, breastfeeding, and close interaction play a significant role in establishing a secure attachment. The emotional connection established during these early interactions has a significant impact on the child’s social and cognitive development.

The advice to permit skin-to-skin contact hinges largely on the understanding that with proper care measures, the risk of horizontal perinatal transmission is minimal, and the advantages surpass the possible dangers of neonatal COVID-19 infection. It is vital to comprehend the rationale behind the existing guidelines for skin-to-skin contact to facilitate effective prenatal parental education, enabling informed shared decision making. Given the minimal risk of transmission and the potential long-term consequences of separation, it is imperative to find a balance between safeguarding infants from the virus and preserving the dynamic connection between mother and child. Healthcare providers can offer recommendations for safe practices that minimize the risk of transmission while promoting meaningful interactions that fulfill the baby’s emotional and developmental needs [62,63,64,65,66].

## 6. Conclusions

In conclusion, our systematic review and meta-analysis focused on assessing the risk of SARS-CoV-2 transmission from infected mothers to neonates during breastfeeding, skin-to-skin contact, and rooming-in. Key findings revealed a low prevalence of SARS-CoV-2 infection among neonates born to infected mothers, with a rate of 1.0%. This suggests a relatively low risk of mother-to-child transmission during the neonatal period.

The sub-analysis indicated consistent proportions of infected neonates across studies, regardless of data availability, for rooming-in, skin-to-skin contact, and exclusive breastfeeding. Despite uncertainties surrounding the exact mode of SARS-CoV-2 transmission through breast milk, the benefits of breastfeeding outweigh the potential risks. Major organizations including the CDC, WHO, and RCOG endorse direct breastfeeding with supportive care measures such as hand hygiene and wearing masks.

The discovery of anti-SARS-CoV-2 antibodies in breast milk has raised the possibility that it may provide protection for neonates. Breast milk, which is rich in bioactive molecules, is a recommended source of nutrition for newborns, even when the mother is ill. Further research is necessary to determine the efficacy of these antibodies and to fully understand their ability to protect against SARS-CoV-2 in the neonatal period.

While acknowledging the limitations, such as the evolving pandemic landscape and the need for research on vaccinated postpartum women, our study provides valuable insights. Addressing gaps and limitations is crucial, and healthcare providers should balance protective measures against potential adverse effects on the emotional and developmental well-being of both mother and child.

In addressing infectious disease outbreaks, maintaining a balanced approach is essential. Research indicates that newborns face a moderate likelihood of contracting COVID-19 from their mothers, underscoring the significance of safeguarding the crucial bond between mother and child. Healthcare practitioners hold a pivotal position in recommending precautionary measures that minimize transmission risks while promoting valuable interactions that nurture the emotional and developmental well-being of neonates and infants.

## Figures and Tables

**Table 1:** Main characteristics of the studies included in this systematic review.

First Author (Year of Publication)	Study Design	Study Period	Country	Number of Mothers with SARS-CoV-2 Infection	Age of Mothers (Years) Mean (±SD)	Asymptomatic COVID-19 Illness, N (%)	Gestational Age (Weeks) Mean (±SD)	Route of Delivery (C/s) N (%)	Number of Neonates
Angelidou et al. (2021) [25]	Multicenter cohort study	1 March to 31 July 2020	USA	250	30.4 (±6.3)	170 (68.0%)	37.9 (±2.6)	113 (45,2%)	255
Congdon et al. (2021) [26]	Case-series study	March to May 2020	USA	70	NR	NR	39.0 (36.3–41.6) **	25 (35.7%)	70
Conti et al. (2021) [29]	Observational study	1 April 2020 to 18 March 2021	Italy	37	31 (17–45) *	10 (27.0%)	39 (32–42) *	18 (48.5%)	37
Dumitriu et al. (2021) [27]	Retrospective cohort study	13 March to 24 April 2020	USA	100	28.5 (24.0–34.0) **	NR	39.0 (37.0–40.0) **	46 (45.5%)	101
Donati et al. (2021) [30]	Prospective cohort study	25 February to 31 July 2020	Italy	525	31.8 (±5.69)	235 (44.8%)	NR	177 (33.7%)	538
Ferazzi et al. (2020) [31]	Retrospective study	1 March to 20 March 2020	Italy	42	32.9 (21-44) *	NR	NR	18 (42.8%)	42
Jiménez et al. (2020) [21]	Prospective observational study	1 March to 31 May 2020	Spain	403	NR	291 (72.2%)	NR	136 (26.0%)	403
Marin Gabriel et al. (2020) [20]	Descriptive study	13 March to 31 May 2020	Spain	242	32.1 (±6.3)	98 (40.5%)	39 (38–40) **	63 (26.0%)	248
Marín Gabriel et al. (2020) [19]	Descriptive study	13 March to 31 May 2021	Spain	42	33.6 (±4.9)	NR	38 (±3.1)	20 (47.6%)	42
Martenot et al. (2021) [33]	Retrospective study	15 March to 24 April 2020	France	26	30.6 (±7.9)	NR	39 (±2)	10 (38.6%)	26
Nayak et al. (2021) [34]	Prospective observational study	1 May to 20 October 2020	India	162	39 (19–41) *	NR	37.5 (25–41) *	103 (63.6%)	165
Pereira et al. (2020) [36]	Retrospective case series study	14 March to 14 April 2020	Portugal	22	34 (19–43) *	11 (50.0%)	38 (31–41) *	4 (18.2%)	22
Ronchi et al. (2020) [32]	Prospective, cohort study	19 March to 2 May 2020	Italy	61	32 (28–36) **	34 (55.7%)	39 (35–41) **	15 (24.6%)	62
Salvatore et al. (2020) [28]	Observational cohort study	22 March to 17 May 2020	USA	78	NR	20 (25.6%)	38 (27–41) *	36 (43.9%)	82
Sánchez García et al. (2021) [22]	Prospective case-control study	April to July 2020	Spain	37	33.9 (±5.4)	16 (43.2%)	39.1 (±1.8)	10 (27.0%)	37
Sánchez-Luna et al. (2021) [23]	Prospective cohort study	8 March to 26 May 2020	Spain	497	33	245 (49.3%)	49 (±3.7)	164 (33.0%)	503
Shlomai et al. (2021) [35]	Observational cohort study	5 March to 30 May 2020	Israel	53	29.7 (±7.3)	40 (75.5%)	39 (±1.0)	10 (18.9%)	55
Solis-Garcia et al. (2021) [24]	Prospective cohort study	15 March to 17 August 2020	Spain	73	34 (27–37) **	32 (43.8%)	38 (37–40) **	26 (35.6%)	75

* Median (min–max); ** Median (IQR); NR, not reported.

**Table 2:** Rate of infected neonates and the practices used by mothers.

First Author (Publication Year)	Number (%) of Infected Infants	Skin-to-Skin, N (%)	Early Separation, N (%)	Rooming-in with Mother, N (%)	Exclusive Breastfeeding, N (%)	Breastfeeding (Exclusive or Complementary), Ν (%)
Angelidou et al. (2021) [25]	6 (2.7%)	NR	NR	167 (66.8%)	152 (66.8%)	230 (90.2%)
Congdon et al. (2021) [26]	0 (0.0%)	NR	33 (47.1%)	33 (47.1%)	21 (30.0%)	33 (47.1%)
Conti et al. (2021) [29]	1 (2.7%)	NR	37 (100.0%)	NR	1 (2.7%)	10 (27.0%)
Dumitriu et al. (2021) [27]	0 (0.0%)	NR	NR	82 (81.2%)	41 (40.6%)	91 (90.1%)
Donati et al. (2021) [30]	18 (3.4%)	NR	279 (51.9%)	379 (72.2%)	ΝR	428 (79.6%)
Ferazzi et al. (2020) [31]	3 (7.1%)	NR	NR	NR	11 (26.2%)	NR
Jiménez et al. (2020) [21]	6 (1.5%)	251 (62.3%)	NR	NR	249 (61.8%)	NR
Marin Gabriel et al. (2020) [20]	13 (5.2%)	NR	NR	NR	103 (41.5%)	179 (72.2%)
Marín Gabriel et al. (2020) [19]	0 (0.0%)	NR	37 (88.1%)	NR	6 (14.3%)	19 (45.3%)
Martenot et al. (2021) [33]	1 (3.9%)	10 (38.5%)	NR	NR	11 (42.0%)	23 (88.5%)
Nayak et al. (2021) [34]	9 (5.5%)	NR	NR	138 (83.4%)	NR	125 (75.8%)
Pereira et al. (2020) [36]	0 (0.0%)	NR	NR	13 (59.1%)	11 (50.0%)	17 (77.3%)
Ronchi et al. (2020) [32]	3 (4.8%)	NR	7 (11.3%)	55 (87.1%)	45 (72.6%)	59 (95.2%)
Salvatore et al. (2020) [28]	0 (0.0%)	NR	NR	68 (82.4%)	NR	64 (78.0%)
Sánchez García et al. (2021) [22]	0 (0.0%)	NR	NR	NR	NR	NR
Sánchez-Luna et al. (2021) [23]	18 (3.6%)	252 (50.0%)	NR	264 (52.3%)	272 (54.1%)	393 (78.1%)
Shlomai et al. (2021) [35]	0 (0.0%)	NR	47 (85.5%)	NR	NR	41 (74.5%)
Solis-Garcia et al. (2021) [24]	1 (1.3%)	51 (68.0%)	NR	NR	48 (64.0%)	55 (73.3%)

NR, not reported.

## Data Availability

The articles’ data supporting this systematic review are from previously reported studies and datasets, which have been cited. The processed data are available in Table 1 and the reference list. Further information can be requested from the corresponding author.

## Public Involvement Statemen

No public involvement in any aspect of this research.

## Guidelines and Standards Statement

This manuscript was drafted against the PRISMA 2020 Statement.
